# Supplementary material for: The causal relationship between the human gut microbiota and pyogenic arthritis: a Mendelian randomization study
Source: Front Cell Infect Microbiol. 2024 Nov 26;14:1452480. doi: 10.3389/fcimb.2024.1452480 (PMC11629706; doi:10.3389/fcimb.2024.1452480)
Supplement: Supplementary file 1 [file DataSheet1.zip › mendelian test/ebi-a-GCST90027780.csv_leave_one_out.pdf]

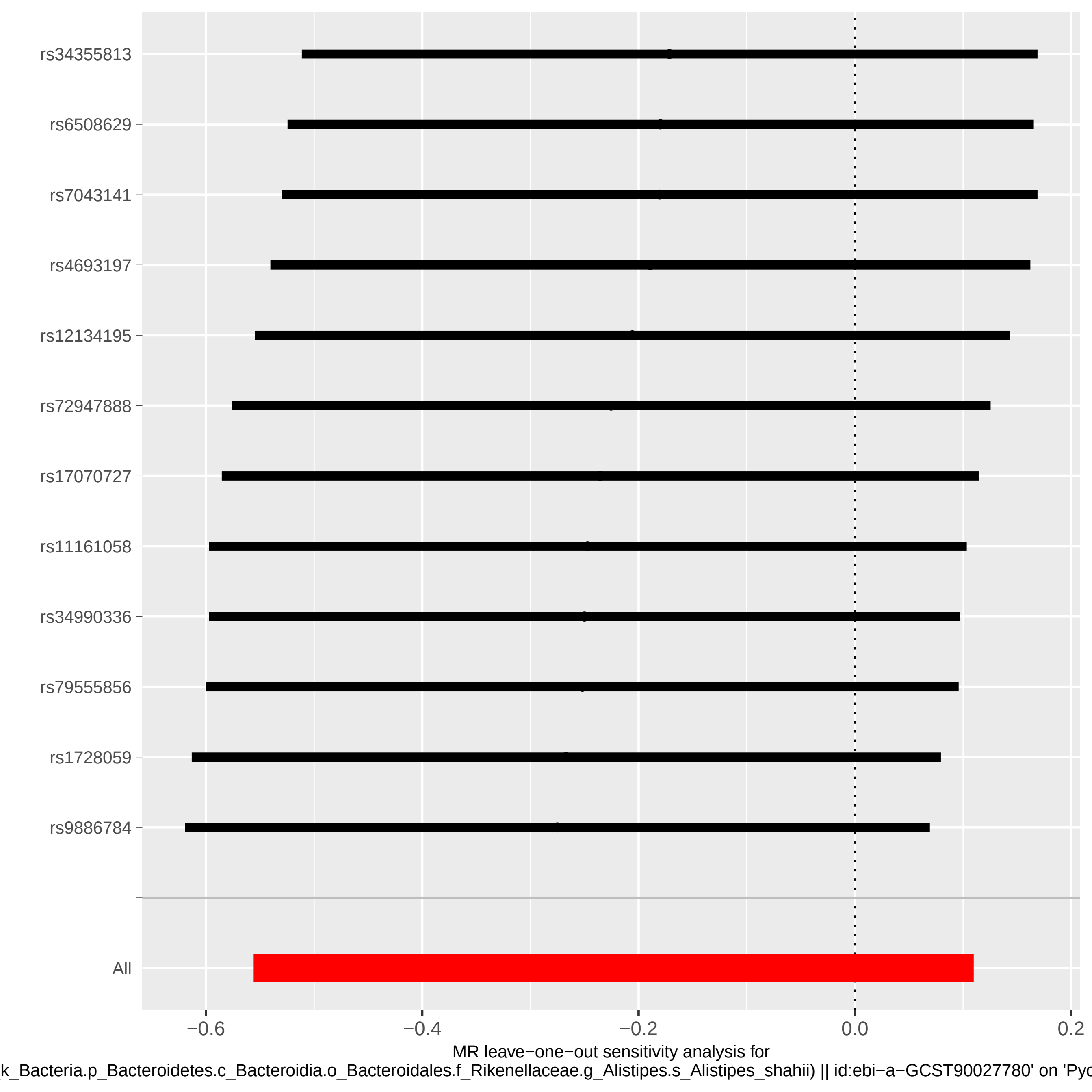
rs34355813
rs6508629
rs7043141
rs4693197
rs12134195
rs72947888
rs17070727
rs11161058
rs34990336
rs79555856
rs1728059
rs9886784
All
−0.6
−0.4
−0.2
0.0
0.2
MR leave−one−out sensitivity analysis for
k_Bacteria.p_Bacteroidetes.c_Bacteroidia.o_Bacteroidales.f_Rikenellaceae.g_Alistipes.s_Alistipes_shahii) || id:ebi−a−GCST90027780' on 'Py
